# Application of a Quantitative POCT-FIT Based on Near-Infrared Fluorescence Immunochromatography in Population-Based Colorectal Cancer Screening: A Comparative Evaluation with a Centralized Testing System

**DOI:** 10.3390/diagnostics16193087

**Published:** 2026-09-24

**Authors:** Fang-Chao Zhu, Yu-Lin Ku, Jie Pan, Xiao-Guang Zhang, Yao-Gen Shu

**Affiliations:** 1 Wenzhou Central Hospital, Wenzhou 325000, China; zhufangchao85@163.com; 2 Wenzhou Institute, University of Chinese Academy of Sciences, Wenzhou 325000, China; kuyulin@ucas.ac.cn; 3 National Key Laboratory of Infectious Disease Traceability, Early Warning and Intelligent Decision-Making, National Institute for Viral Disease Control and Prevention, China CDC, Beijing 100052, China; zhangxg@ivdc.chinacdc.cn

**Keywords:** quantitative detection technology, near-infrared fluorescence, immunochromatography, fecal occult blood, colorectal cancer screening, decentralized testing

## Abstract

**Background/Objectives**: Fecal occult blood testing (FOBT) is a cornerstone of colorectal cancer (CRC) screening. The current quantitative FOBTs, however, were centralized, which limited their accessibility in preliminary screening implemented by community health. This study aimed to establish a rapid, highly sensitive quantitative FOB-detection method based on near-infrared fluorescence immunochromatography, hereinafter referred to as point-of-care fecal immunochemical testing (POCT-FIT), and to evaluate its performance against a centralized testing system in population-based CRC screening. **Methods**: Anti-human hemoglobin monoclonal antibodies were labeled with near-infrared fluorescent nanospheres (excitation 770 nm; emission 794 nm), and FOB test strips were prepared using immunochromatography. A fluorescence immunochromatography analyzer was used to scan the fluorescence intensities of the test line (T) and control line (C). The ratio of the fluorescence intensity on the T-line to that on the C one (T/C) was calculated at different hemoglobin concentrations, and a standard curve was generated over the span of 50 to 2000 ng/mL. A total of 115 clinical fecal samples were collected for methodological comparison with the commercially available OC-Sensor system (Eiken Chemical Co., Ltd., Tokyo, Japan). In a non-paired CRC screening program implemented by community health in Wenzhou, 3163 participants were assigned to the centralized testing group (OC-Sensor system) and 5211 participants to the POCT-FIT group (near-infrared fluorescence immunochromatography method). A positive threshold was set up at 100 ng/mL. Colonoscopy and histopathological results served as the gold standard. **Results**: The testing results of the two groups for the 115 samples showed that there is a strong linear correlation (*R* = 0.9959) and good agreement between the two methods. In the screening of community health, CRC prevalence was 2.78% (88/3163) in the centralized group (OC-Sensor) and 1.00% (52/5211) in the decentralized one (POCT-FIT). Sensitivities for CRC were 98.9% (95% CI: 95.8–99.9%) and 100% (95% CI: 93.2–100%), respectively, with no statistically significant difference; specificities were 29.2% (95% CI: 27.6–30.8%) and 27.3% (95% CI: 26.1–28.5%), with the centralized group being slightly higher (*p* = 0.001). Positive and negative likelihood ratios were comparable between the two methods. **Conclusions**: The sensitivity of the POCT-FIT method was comparable to that of the OC-Sensor method, and its negative results were associated with a lower probability of missed CRC, which may help reduce medical insurance expenditure for CRC treatment. POCT-FIT is simple to operate and portable, and the price of the disposable immunochromatographic test strip is much lower than that of the turbidimetric method (centralized testing system). Thus, it meets the practical needs of settings with low daily testing numbers (such as preliminary community health or resource-limited and sparsely populated areas) and serves as an effective complement to centralized testing.

## 1. Introduction

Colorectal cancer (CRC) is the third-most common malignancy worldwide, with approximately 1.93 million new cases and 0.93 million deaths in 2020 [1]. China faces a similarly heavy burden, with CRC incidence continuing to rise [2]. Fecal occult blood testing (FOBT) is recommended as a preliminary screening method by screening guidelines due to its non-invasiveness, simplicity, low cost, and high patient acceptability [3,4]. Large-scale prospective cohort studies have confirmed that two or more FOBT screenings reduce CRC mortality by 44% [5], and fecal hemoglobin concentration is one of the strongest predictors of future CRC detection (adjusted OR = 9.4) [6], further reinforcing the central role of FOBT in CRC screening.

Traditional qualitative FOBT methods, such as colloidal gold immunochromatography or guaiac-based tests, are convenient, but there are notable limitations—subjective result interpretation and high false-positive rates in population screening, leading to unnecessary colonoscopy referrals [7]. The Cochrane systematic review (published in 2022) clearly demonstrated that quantitative fecal immunochemical testing (FIT) significantly outperforms traditional qualitative methods in diagnostic accuracy for CRC [8]. A recent multicenter diagnostic trial in China (2618 outpatients) showed that quantitative FIT at a 20 ng/mL cutoff achieved 100% sensitivity for CRC, whereas three qualitative FIT methods achieved only 66.7–83.3% [9]. A large-scale screening study in Haining County, Zhejiang Province (2021–2023) also confirmed that quantitative FIT provides superior screening performance and lower unit cost than the qualitative one [10]. Thus, it seems irreversible that the qualitative FIT will be replaced by the quantitative one.

The most international widely used quantitative FIT system is the OC-Sensor platform (Eiken Chemical Co., Ltd., Tokyo, Japan), which employs latex agglutination immunoturbidimetry and offers good performance. However, this platform requires expensive automated analyzers operated by trained personnel, a centralized laboratory, and cold-chain transport of fecal samples collected from different community health centers. The high instrument cost and per-test expense severely limit its deployment in community health and resource-limited settings [11]. Although international guidelines have begun recommending quantitative FIT for CRC referral in preliminary screening [12], to expand the quantitative preliminary screening is still blocked by high cost. Therefore, developing a simple, economical, and on-site operable (“decentralized”) quantitative FOBT technology (POCT) is urgent for expanding of CRC screening.

Near-infrared fluorescence immunochromatography, developed by us, offers several advantages in quantitative POCT applications [13,14,15], such as high sensitivity, because the emission light is lower in energy and avoids the autofluorescence of biological macromolecules, and super stability due to the lack of quenching, etc. In this study, we established a novel rapid and highly sensitive quantitative FOB-detection method based on this technology and systematically evaluated its performance in clinical sample comparison and community health-based screening in Wenzhou, and provided a practical decentralized POCT-FIT for preliminary CRC screening.

## 2. Materials and Methods

### 2.1. Reagents and Instruments

The near-infrared fluorescence immunochromatography platform (including the WZBIOG-2022 fluorescence immunochromatography analyzer and its matching FOB test strips) was developed by our research team and manufactured under standardized quality control by Wenzhou Baiouji Technology Co., Ltd. (Zhejiang, China) according to our technical specifications. EDC and human hemoglobin were purchased from Sigma-Aldrich (St. Louis, MO, USA). Trehalose was obtained from Yuanye Bio (Shanghai, China). Herpes buffer was purchased from Gibco (Life Technologies Corporation, Carlsbad, CA, USA). Fluorescent pads were obtained from Hangzhou Biokai Lab Biotechnology Co. (Hangzhou, China). PBS and other common buffers were prepared in-house. FOB coating monoclonal antibodies, FOB labeling monoclonal antibodies, near-infrared fluorescent nanospheres and fluorescent label diluent, goat anti-mouse IgG polyclonal antibodies, diluent, blocking solution, and suspension solution were purchased from Beijing Runbo Fude Biotechnology Co. (Beijing, China). Nitrocellulose membranes (CN140) were obtained from Sartorius (Göttingen, Germany). Backing cards, absorbent paper, sample pads, a continuous membrane strip dispenser, and a microcomputer automatic cutting machine (Z2000) were purchased from Shanghai Jieyi Biotechnology Co. (Shanghai, China). Test cassettes were purchased from Ningbo Liudeng Medical Technology Co. (Ningbo, China). A total of 115 clinical fecal samples were obtained from the Department of Gastroenterology, Wenzhou Central Hospital. The comparison reagent was the OC-Sensor quantitative detection system (Eiken Bio-Technology (China) Co., Shanghai, China).

### 2.2. Preparation of Fluorescence Immunochromatography Test Strips

Membrane preparation: The nitrocellulose membrane was stuck to a backing card of polyvinyl chloride and was relaxed at 45–65% relative humidity for 2 h before use.

Membrane coating solution preparation: The coating solution consisted of 10 mM PBS, 5% trehalose, pH = 7.0 ± 0.1. The FOB coating antibody was diluted to 1 mg/mL and goat anti-mouse IgG polyclonal antibody to 0.5 mg/mL. The solution was sprayed onto the nitrocellulose membrane at 1.0 µL/cm using a dispenser to form the test line (T-line) and control line (C-line), followed by drying.

Near-infrared FOB fluorescent label conjugation: A total of 100 µL of carboxylated near-infrared fluorescent nanospheres were activated with EDC for 30 min, centrifuged, and resuspended in 50 mM Herpes (pH 7.0). Then, 100 µg of FOB labeling antibody was added and reacted at room temperature for 1 h. After centrifugation, the pellet was resuspended in 100 µL blocking solution and incubated for 1 h at room temperature. Following another centrifugation, the pellet was resuspended in 100 µL suspension solution and stored at 4 °C.

Fluorescent pad preparation: The near-infrared FOB fluorescent label was diluted 300-fold with fluorescent label diluent, mixed thoroughly, uniformly coated onto the fluorescent pad, and dried.

Card assembly: Absorbent paper was attached to the shorter end of the membrane support card, overlapping the membrane by 1–2 mm. On the opposite end, the fluorescent pad and sample pad were sequentially attached below the membrane, with 1–2 mm overlaps. The sample pad was flush with the lower edge of the support card as shown in Figure 1.

Strip cutting and cassette assembly: The assembled card was cut into 3 mm wide strips using an automatic cutter and placed into test cassettes.

### 2.3. Standard Curve Establishment

FOB reference standards were serially diluted to concentrations of 50–2000 ng/mL. Then, 60 µL of each dilution was added to the sample well of the test cassette. After 15 min of incubation at room temperature, the cassette was inserted into the fluorescence immunochromatography analyzer to scan the fluorescence intensities of the T-line and C-line (as shown in Figure 3). The T/C ratio was calculated and plotted against FOB concentration as shown in Figure 4. The standard (working) curve was imported into the fluorescence immunochromatography analyzer via QR code.

### 2.4. Clinical Sample Comparison

Fecal samples from patients with gastrointestinal bleeding and healthy individuals were collected. Each sample was divided into two aliquots: one was processed with the OC-Sensor fecal diluent and sent to the testing facility (Eiken Bio-Technology (China) Co.); the other was processed with our developed diluent and tested using our developed reagents and instrument. After 15 min of chromatography, the cassette was inserted into the fluorescence analyzer for FOB concentration measurement. The 115 valid results from both methods were plotted in a two-dimensional scatter plot as shown in Figure 5, and the linear correlation coefficient was calculated; Bland–Altman analysis with SD was also performed [16].

### 2.5. POCT-FIT and OC-Sensor Screening (Non-Paired Design)

The FIT results and corresponding colonoscopy and pathological findings from the 2024–2025 CRC screening program for the high-risk population (aged 50–74) in Wenzhou were collected. The screening protocol was as follows: all participants underwent FIT and a CRC risk-assessment questionnaire. Those with positive FIT (test value ≥ 100 ng/mL) or those assessed as high-risk by the questionnaire were recommended to undergo colonoscopy. Colonoscopies were performed by experienced endoscopists at participating hospitals, with the entire colon examined segment by segment. Suspicious lesions were biopsied and sent for pathological examination. Pathological diagnoses were independently interpreted by two pathologists, with a third senior pathologist arbitrating in cases of disagreement; the final consensus served as the gold standard.

The screening performance (sensitivity, specificity, positive predictive value, negative predictive value, positive likelihood ratio, negative likelihood ratio) of the centralized quantitative testing system (OC-Sensor) and the POCT-FIT quantitative testing system (near-infrared fluorescence immunochromatography method) for CRC was analyzed using SPSS 26.0 (IBM Corp., Armonk, NY, USA). A test value ≥ 100 ng/mL was defined as positive. Written informed consent was obtained from all participants prior to enrollment. The threshold of 100 ng/mL was determined by ROC curve analysis (AUC = 0.825, 95% CI: 0.773–0.877, *p* < 0.001); sensitivity and specificity at different thresholds are presented in Section 3.5.

## 3. Results

### 3.1. Near-Infrared Fluorescence Detection

The near-infrared fluorescent nanospheres possess activated carboxyl groups (−COOH) that can condense with amino groups (−NH_2_) of labeled antibodies to form amide bonds. The excitation and emission wavelengths are 770 nm and 794 nm, respectively. The labeled complex can be stored stably in a dry state.

### 3.2. Immunochromatography Test Strip

The immunochromatography test strip consisted of a backing card, absorbent paper, nitrocellulose membrane, a fluorescent pad, and a sample pad (Figure 1).

After the sample is applied to the sample pad, it migrates via capillary action toward the fluorescent pad and the nitrocellulose membrane. The fluorescently labeled antibodies in the pad bind to the target to form a complexes that will be captured by immobilized antibodies at the T-line, forming captured antibody–target–fluorescent label complexes (T-line). Excess fluorescent labels will be captured by goat anti-mouse IgG at the C-line. Upon excitation, the fluorescent labels emit a signal that will be detected by the fluorescence analyzer.

The near-infrared fluorescence scanner used an LED as the excitation source (Figure 2). After passing through a filter, a 770 nm parallel beam was reflected by a splitter and focused onto the sample by a confocal lens. The photons emitted from the sample were collected by the same lens; the 794 nm parallel beam passed through the splitter and the emission filter, and finally was detected by the detector. This design was according to the “confocal mechanism”.

**Figure 2:**
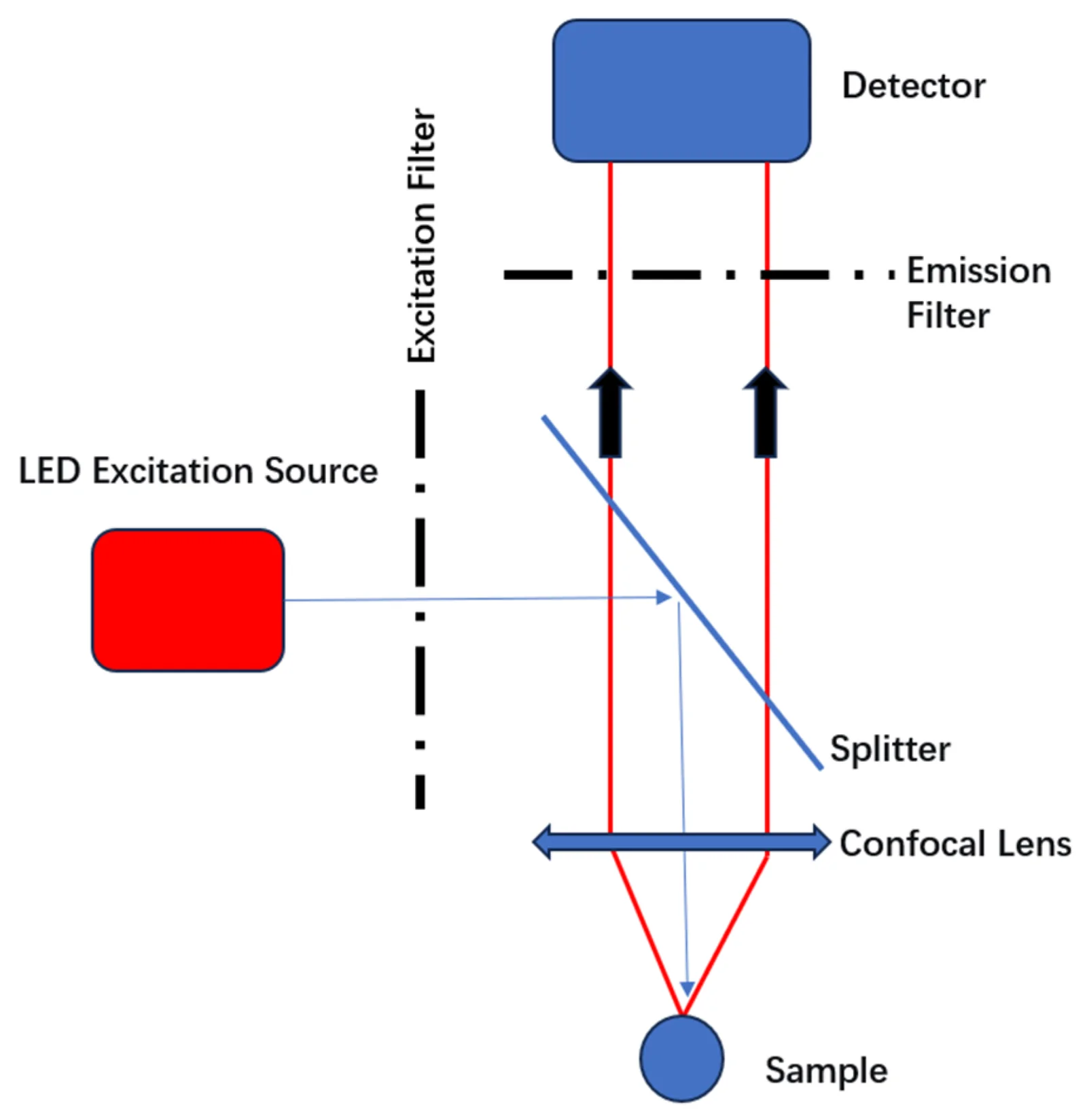
Schematic diagram of the near-infrared fluorescence immunochromatography analyzer with confocal principle. The system consists of an LED excitation source, an excitation filter, a beam splitter, a confocal lens, a sample stage, an emission filter, and a detector. The excitation light (770 nm) is focused onto the test strip, and the emitted near-infrared fluorescence (794 nm) is collected by the detector for signal quantification.

As the stepper motor drove the sample stage, the intensity of the emission light was scanned from the absorbent-paper end to the sample-loading end of the nitrocellulose membrane. A fluorescence intensity profile (including two pulses) was obtained against time as shown in Figure 3, where the time is tightly coupled with the distance along with the strip by the stepper motor.

**Figure 3:**
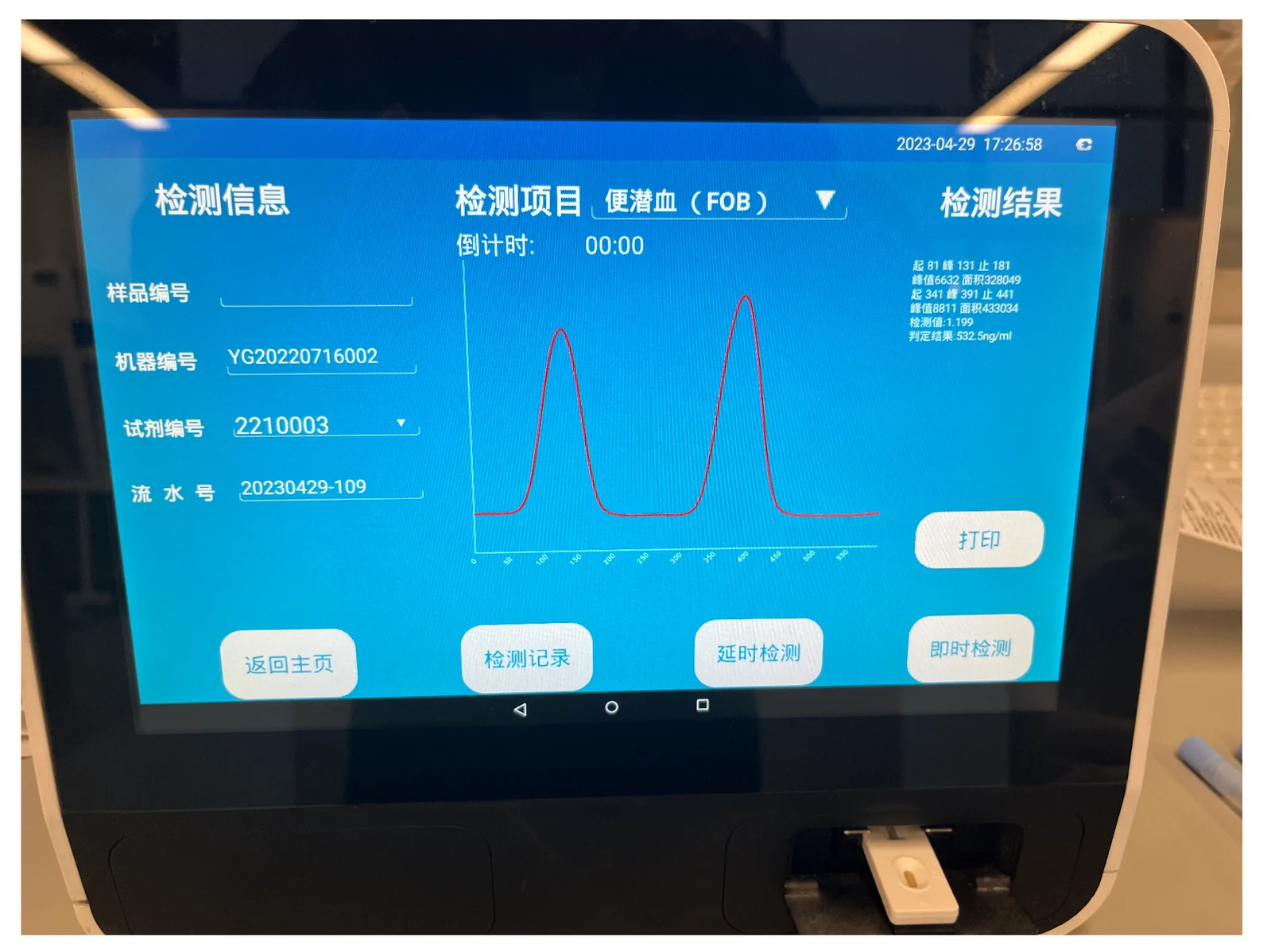
Scanning profile of FOB fluorescence intensity (red). The right pulse is the T-line; the left pulse is the C-line.

### 3.3. Standard Curve

The fluorescence intensities of the T- or C-line were positively correlated with the amount of fluorescently labeled complexes that were captured by the antibodies immobilized on the corresponding line. After each diluted reference standard sample was tested, the T/C value at each concentration was used to generate the working curve for the span of FOB concentration (Figure 4). The advantage of using the ratio of fluorescence intensities between the T-line and the C-line (T/C) is that it avoids interference caused by the detection environment and eliminates the need for calibration, which is otherwise required when measuring absolute fluorescence intensity values.

In the span of FOB concentration—50–2000 ng/mL, the four-parameter logistic fit was used.

(1) $$y=\frac{a-d}{1+{\left(x/c\right)}^{b}}+d$$

**Figure 4:**
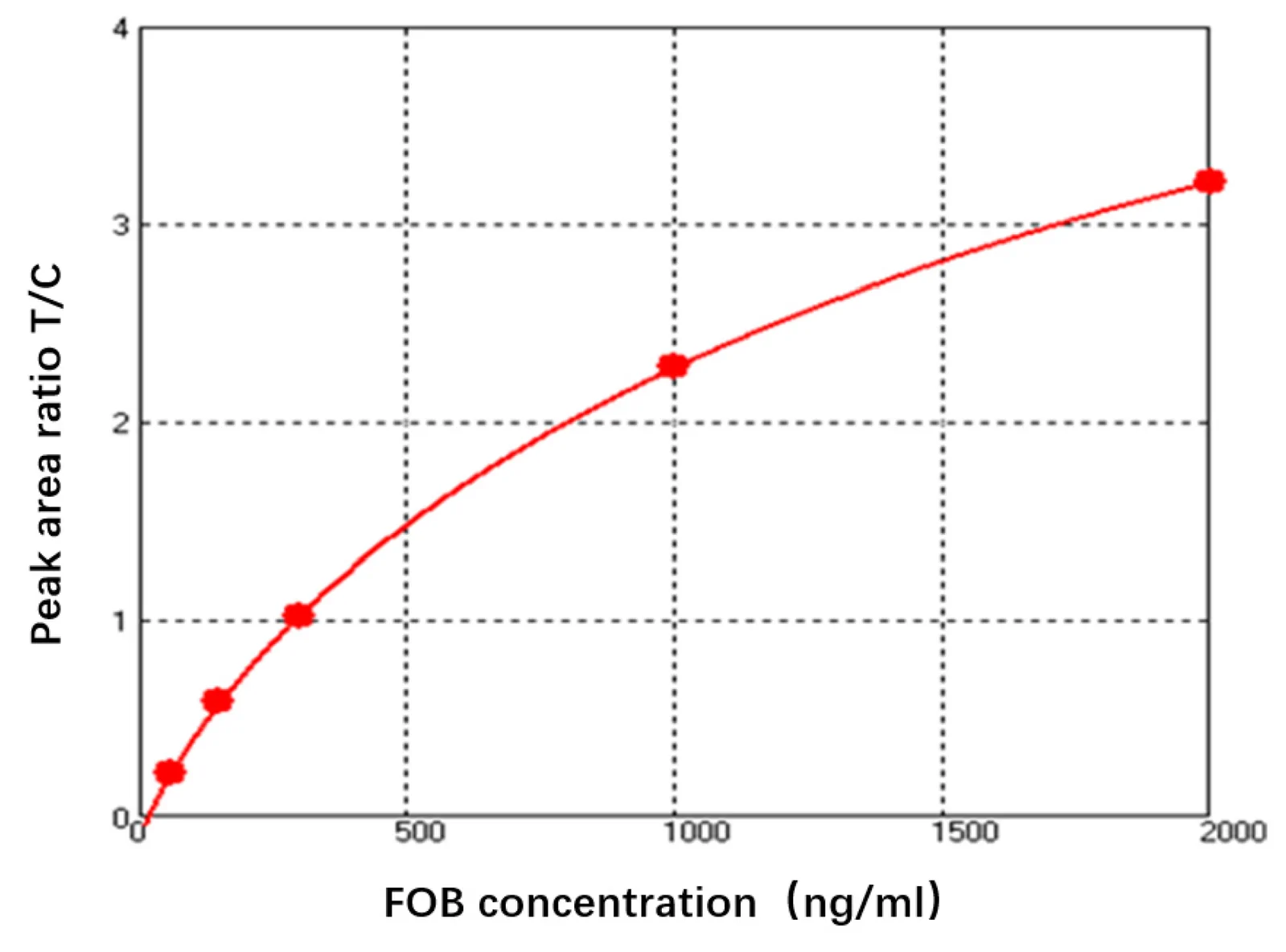
The working curve of the near-infrared fluorescence method. The four-parameter logistic fit (see Equation (1)) yielded the following estimates: upper asymptote a = 6.674, lower asymptote d = −0.1286, slope b= −0.8179, and half-maximal effective concentration c = 2092. The experimental data showed excellent fit to the curve (*R* ≈ 1).

The fitted parameters were: upper asymptote *a* = 6.674; lower asymptote *d* = −0.1286; slope *b* = −0.8179; and half-maximal effective concentration *c* = 2092. The experimental data showed excellent fit to the curve (*R* ≈ 1.0).

### 3.4. Comparison Resulted from Clinical Samples

A total of 115 clinical fecal samples were tested. Each sample was split for parallel testing by both methods. The results showed that the two methods are in “perfect equality” with *R* = 0.9959 as shown in Figure 5a. Bland–Altman analysis [16] of the difference between the two measurements with a 95% limit of agreement is displayed in Figure 5b, providing a highly informative visual summary.

**Figure 5:**
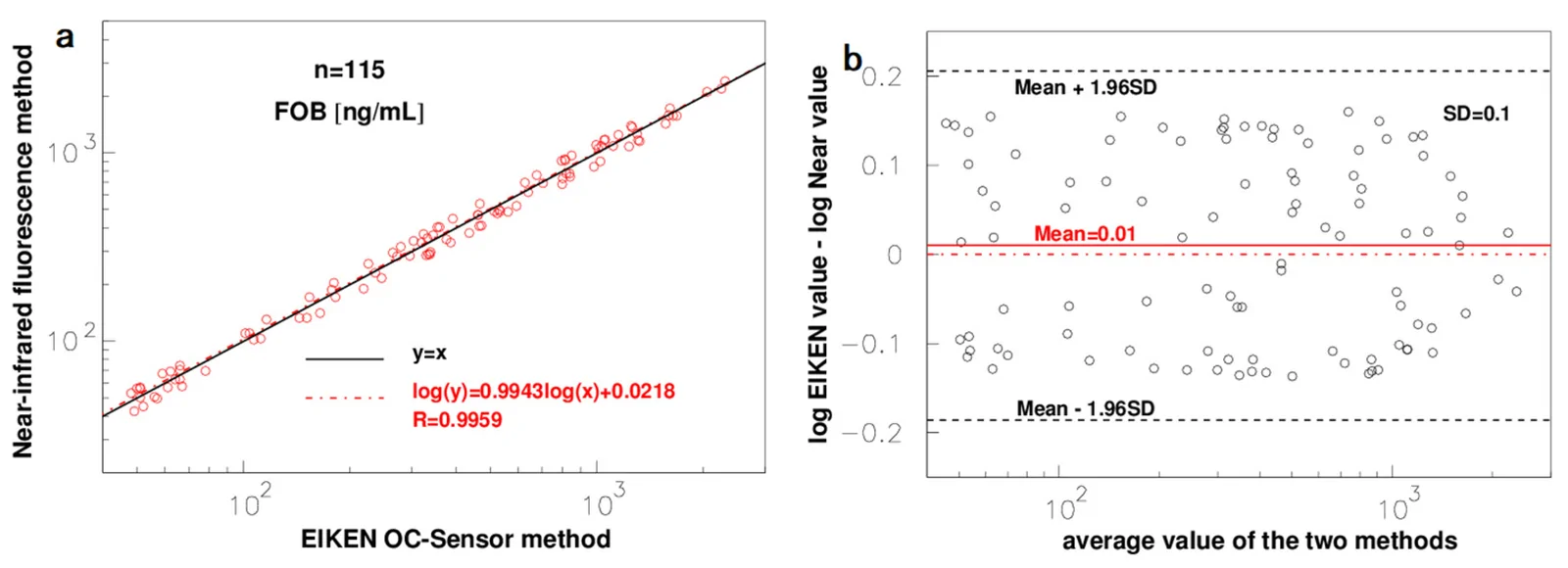
(**a**) A scatter plot showing the measurements from POCT-FIT (y-axis) versus OC-Sensor (x-axis) is presented, along with the y = x line of equality. The fitted line is also plotted here with R = 0.9959. Results show good agreement between the two methods. (**b**) A “difference-versus-mean” scatter plot was generated by plotting the difference between the two methods (y-axis) against their mean (x-axis), clearly revealing inconsistencies that were not apparent in (**a**). The plot incorporates the bias between the two methods (estimated by the mean difference) and the 95% limits of agreement (mean ± 1.96 SD), providing a highly informative visual summary. In such studies, as the true value is unknown, the mean of the measurements from the two methods serves as the best estimate.

### 3.5. The Positive Threshold and Community Health-Based Screening

#### 3.5.1. Determination of the Positive Threshold

Based on the large-scale screening data from community health centers, the optimal positive threshold for POCT-FIT was determined by ROC curve analysis. The AUC was 0.825 (95% CI: 0.773–0.877, *p* < 0.001). Sensitivity and specificity at different thresholds were further compared (Table 1). At 100 ng/mL, sensitivity was 100% with a Youden index of 0.279. Though the specificity will be improved to 77.1% if the threshold is set to 500 ng/mL, the sensitivity will decrease to 76.9%. Because the sensitivity plays a dominant role in the evaluation of the quality of preliminary screening, 100 ng/mL was selected as the positive threshold.

#### 3.5.2. Colonoscopy Verification and Detection Rate

In the analysis population, the OC–Sensor group had 6158 FIT-positive participants, of whom 3163 underwent colonoscopy (adherence rate 51.4%), and 88 CRC cases were confirmed (detection rate 2.78%, 88/3163). The POCT–FIT group had 9833 FIT-positive participants, of whom 5211 underwent colonoscopy (adherence rate 53.0%), and 52 CRC cases were confirmed (detection rate 1.00%, 52/5211) (Table 2). The significant difference in detection rates between the two groups suggests different baseline risks of different populations.

#### 3.5.3. Comparison of Screening Performance

Sensitivity: No statistically significant difference was observed between the two groups (*p* > 0.05). Both methods effectively identified CRC, with the POCT-FIT group achieving 100% sensitivity.

Specificity: The centralized testing group showed higher specificity than the POCT-FIT group (*p* ≤ 0.001).

Likelihood ratios: The positive likelihood ratios (LR+) and negative likelihood ratios (LR−) for CRC were comparable between the two methods, indicating no substantial difference in their ability to confirm or exclude CRC.

Positive predictive value (PPV): The centralized testing group showed higher PPV than the POCT-FIT group.

## 4. Discussion

Fluorescence immunochromatography enables quantitative analysis by measuring the fluorescence intensity ratio of T to C. Most fluorescence immunochromatography quantitative assays, however, use ultraviolet excitation (higher energy), which induces autofluorescence from proteins or nucleic acids, thereby increasing background noise and decreasing sensitivity. In this study, we used near-infrared fluorescent nanospheres (excitation 770 nm/emission 794 nm) as labels. The emission is in the near-infrared region (lower energy), which avoids the autofluorescence of biological macromolecules (mainly in the visible region) and significantly improves the ratio of signal-to-noise (sensitivity). The size of the label is sufficiently small so that the steric hindrance effects in the interaction between antigen and antibody are eliminated and there is no fluorescence quenching; thus, the new method significantly enhances the measuring stability and span. This strategy has been validated in NT-proBNP, HIV p24 antigen, cardiac biomarkers, and COVID-19 detections [13,14,15]. The present study extends this technology to FOB quantification, demonstrating the versatility and translational value of this technological platform.

Methodologically, the near-infrared fluorescence immunochromatography platform showed excellent quantitative concordance with the OC-Sensor system in 115 clinical samples (*R* = 0.9959). Despite differences in detection principles (immunochromatography vs. latex agglutination immunoturbidimetry) and operational modes (POCT-FIT vs. centralized laboratory automation), our method achieved quantitative values consistent with the widely used reference system, suggesting reliable quantitative accuracy and providing a preliminary methodological basis for clinical screening applications.

In the CRC screening program for high-risk populations in Wenzhou, this study employed a non-paired design to compare the diagnostic performance of two quantitative FIT methods—a centralized testing model (OC-Sensor system) and a POCT-FIT model (a near-infrared fluorescence immunochromatographic system developed in this study) as shown in Table 3. The main findings were as follows: (1) both methods showed high sensitivity for CRC–98.9% (95% CI: 95.8–99.9%) for the centralized group and 100% (95% CI: 93.2–100%) for the POCT-FIT group, with no statistically significant difference; (2) the centralized group showed higher specificity (29.2% vs. 27.3%, *p* = 0.001) than the decentralized method; (3) LR+ and LR− of the centralized method vs that of the decentralized one were approximately 1.39 vs. 1.38 and 0.04 vs. 0, respectively.

Centralized testing systems demonstrated approximately 2% higher specificity, a difference that was statistically significant. This disparity is likely attributable primarily to the testing model: central laboratories operate under standardized environmental controls, professional operational protocols, and rigorous quality control systems, which help minimize false-positive results caused by on-site factors such as operator variability and fluctuations in reagent storage conditions [17]. Previous studies have shown that the false-positive rates for immunochemical FOBT in the institutions participating in laboratory quality management programs were significantly lower than that in those without laboratory quality management programs (2.35% vs. 5.60%), suggesting that standardized quality control plays an important role in reducing false positives [17]. In addition, differences in positive rates across various FIT platforms can reach up to 4-fold, indicating that the operating environment and the standardization of quality control have a significant impact on testing performance [18].

Low specificity is an inherent limitation of FIT screening, as fecal hemoglobin may originate from multiple sources. These include: (1) lower gastrointestinal bleeding (e.g., CRC, adenomas including those precancerous and benign, inflammatory bowel disease, diverticular disease, ischemic colitis, infectious enteritis, angiodysplasia, or intestinal ulcers); (2) upper gastrointestinal bleeding (e.g., gastric or duodenal ulcers, gastritis, or esophageal varices); (3) anorectal bleeding (e.g., hemorrhoids or anal fissures); (4) medication-related bleeding (e.g., anticoagulants, antiplatelet agents, or NSAIDs); and (5) other contaminating sources (e.g., menstrual blood or urinary tract bleeding). However, colonoscopy classifies only CRC (here, excluding precancerous adenomas) as true positive. In this study, the specificities of POCT-FIT and OC-Sensor were 27.3% and 29.2%, respectively; while these figures reflect reality, they also imply that approximately 70% of screening participants testing positive do not have CRC. Specifically, out of 100,000 screening participants testing positive, the two methods would yield approximately 72,700 and 70,800 false-positive cases, respectively, potentially leading to a large number of unnecessary colonoscopies. However, in Zhejiang Province, China, the cost of a colonoscopy is only approximately 400 RMB (about 55 USD), whereas the median treatment cost for CRC is typically 300,000 RMB (about 40,000 USD). Therefore, from a holistic perspective, the costs associated with false-positive screening results may be far lower than the medical costs and loss of life resulting from false-negative results. Under the current healthcare policy framework, it may be more pragmatic to moderately increase sensitivity to reduce missed diagnoses. Naturally, reducing the false-positive rate of screening remains a direction for our future research. Encouragingly, researchers such as Hassen C. have introduced AI (artificial intelligence)-based computer-aided diagnosis (CADx) to evaluate the benefits, drawbacks, accuracy, and feasibility of the traditional “resect-and-discard” strategy for colorectal polyps in clinical practice [19]. We can draw upon their experience and utilize AI technology to adjust positive screening thresholds for specific regional populations in a timely manner, based on local lifestyle habits and historical screening and colonoscopy data. Additional measures include incorporating options for hemorrhoids and anal fissures into risk-stratification questionnaires, advising against the consumption of spicy foods in the 24 h prior to sampling, and including other biomarkers. These preliminary strategies require validation through comprehensive health-economic evaluations.

In terms of overall diagnostic performance, the Youden indexes for the two methods were 0.281 and 0.273, respectively. The Youden index (sensitivity + specificity − 1) is independent of prevalence and serves as a composite measure of overall accuracy; the closer its value is to 1, the higher diagnostic accuracy is. In this study, the Youden indexes for both methods were relatively low (approximately 0.28), reflecting the inherent limitations of FOB testing as a preliminary screening tool: the coexistence of high sensitivity and low specificity means that a single FIT cannot simultaneously ensure the accurate identification of patients and the accurate exclusion of non-patients. These results provide a statistical explanation for why individuals with a positive FIT result must undergo colonoscopy to confirm the diagnosis [16]. More importantly, the difference in Youden indexes between the two methods was only 0.008, indicating that the POCT-FIT method does not differ substantially from centralized one in terms of overall diagnostic performance; suggesting that decentralized testing may not compromise diagnostic accuracy.

The core value of FOBT as a preliminary screening tool for CRC lies in its sensitivity approaching 100% and an LR− near 0; a low false-negative rate implies a low probability of missing a CRC diagnosis. Both methods performed consistently regarding this key index, demonstrating that negative results offer reliable exclusionary value under either centralized or POCT-FIT methods. This finding aligns with conclusions from recent domestic and international studies on FIT screening [8,9]. Meanwhile, an LR+ of approximately 1.39 reflects the inherent limitations of FOB: as there are at least four sources of FOB other than CRC and precancerous adenomas, a positive result alone is insufficient to definitively diagnose the disease [20].

It is worth noting that there was a significant difference in detection rates between the two methods (2.78% for the centralized testing method vs. 1.00% for the decentralized testing one). The primary reason for this difference is that the screening participants in the two groups originated from different screening areas (Yueqing County and Ruian County), where baseline risks may have differed; consequently, the detection rates cannot be directly compared, nor should this be used to determine which testing method is superior. The positive predictive values (PPV) also differed between the methods (3.84% vs. 1.37%); however, since PPV is influenced by prevalence, direct intergroup comparison is inappropriate. In contrast, sensitivity, specificity, likelihood ratios, and the Youden index are independent of prevalence and are suitable for comparison between methods. This study demonstrated similar performance across these core indexes (sensitivity: 98.9% vs. 100%; specificity: 29.2% vs. 27.3%; positive likelihood ratio: 1.39 vs. 1.38), suggesting that the diagnostic efficacy of the two methods may be comparable. Nevertheless, these preliminary findings require validation through paired or randomized controlled studies. Encouragingly, another research team is currently preparing a FOB cohort study based on these two methods.

It must be pointed out that this study has the following limitations. First, the use of a non-paired design resulted in differences in baseline characteristics and CRC prevalence between the two study populations. Although core indexes (sensitivity, specificity, likelihood ratios, and Youden index) are generally considered independent of prevalence, this mathematical independence relies on the assumption that the operating characteristics (ROC curves) of the testing methods remain constant across different populations. In this study, both methods employed an identical positive threshold (100 ng/mL) and consistent colonoscopy confirmation criteria; thus, the comparison of core indexes between the groups remains valid. However, future studies should utilize paired or randomized designs within the same population to completely eliminate the confounding effects of population differences. Second, the screening design involved performing colonoscopies only on individuals who tested positive via FIT or were identified as high-risk based on the questionnaire, introducing partial verification bias; specifically, the status of colorectal lesions in individuals who tested negative via FIT and were classified as low-risk by the questionnaire remained unknown, potentially leading to an overestimation of sensitivity and specificity. Nevertheless, since both methods were evaluated within the same screening framework—resulting in similar directions and magnitudes of bias—the validity of the comparison between the groups is robust. Third, this study did not perform stratified adjustments for potential confounding factors such as age, sex, family history, and geographic region; subsequent analyses could further investigate the impact of these factors on the performance of the different testing methods.

## 5. Conclusions

The decentralized (POCT-FIT) and centralized (OC-Sensor) quantitative FIT methods demonstrate comparable sensitivity in detecting CRC—both achieving high levels—and a negative result was associated with a low probability of missed CRC. Youden index analysis suggests that the overall diagnostic accuracy of the two methods may be similar. POCT-FIT utilizes a “real-time sampling and timely testing” approach, ensuring sample freshness while reducing testing costs and turnaround time; this meets the practical needs of settings with low daily testing numbers (such as preliminary community health or resource-limited and sparsely populated areas) and serves as an effective complement to centralized testing. This study provides preliminary evidence for the effective application of decentralized quantitative fecal immunochemical testing in CRC screening and offers valuable experience for the broader adoption of POCT quantitative testing technologies.

## 6. Patents

The fluorescence immunochromatography analyzer (WZBIOG-2022) and the matching FOBT kit (fluorescence immunochromatography method) used in this study have obtained medical device registration certificates from the National Medical Products Administration of China (reagent kit: Zhejiang Medical Device Approval 20232402077; analyzer: Zhejiang Medical Device Approval 20232222075). Related technologies have been patented. Y.S. (Shu Yao-Gen) is the technical inventor.

## Figures and Tables

**Figure 1:**
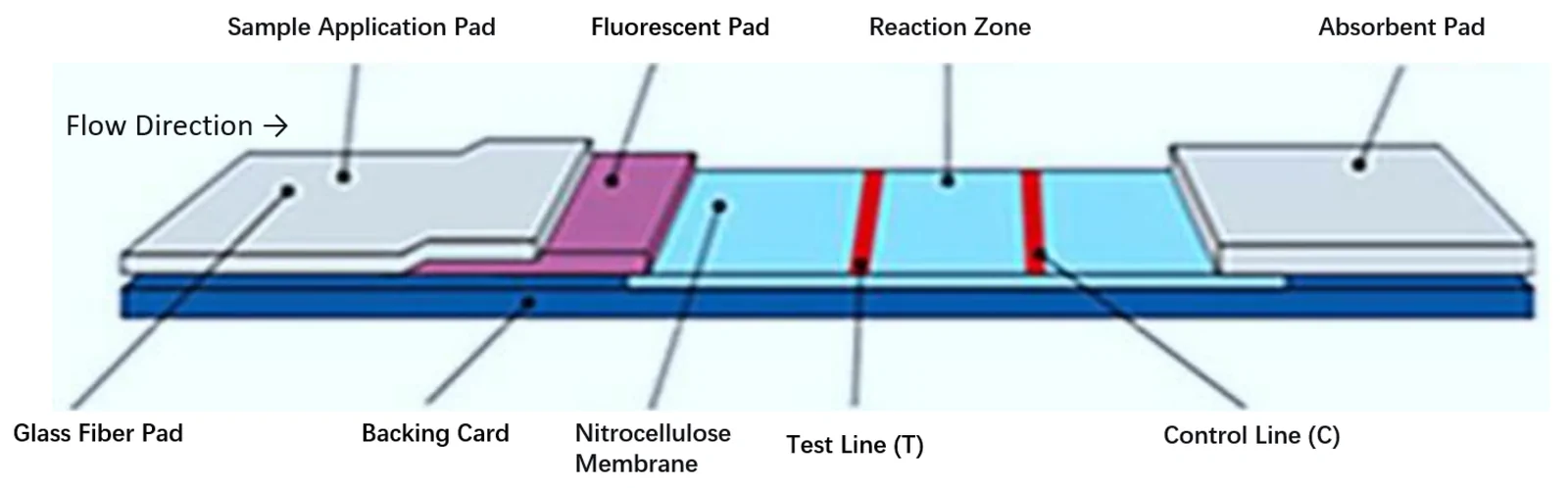
Schematic diagram of the immunochromatographic test strip. The strip consists of a sample application pad, a glass fiber/conjugate pad preloaded with near-infrared fluorescently labeled anti-human hemoglobin antibodies, a nitrocellulose membrane coated with test (T) and control (C) lines, an absorbent pad, and a backing card. The sample migrates from the sample pad through the conjugate pad to the nitrocellulose membrane by capillary action, and the T-line and C-line fluorescence intensities are measured for quantitative analysis.

**Table 1:** Sensitivity and specificity of POCT-FIT at different thresholds.

Threshold (ng/mL)	Sensitivity	Specificity	Youden Index
100.5	100.0%	27.9%	0.279
132.5	94.2%	40.4%	0.346
150.5	90.4%	45.2%	0.356
178.5	88.5%	51.2%	0.397
200.5	88.5%	55.0%	0.435
500.5	76.9%	77.1%	0.540
1000.5	63.5%	85.9%	0.494

**Table 2:** Four-fold tables for both groups.

Group	FIT Result	CRC	Non-CRC	Total
OC-Sensor	Positive	TP = 87	FP = 2177	2264
	Negative	FN = 1	TN = 898	899
POCT-FIT	Positive	TP = 52	FP = 3751	3803
	Negative	FN = 0	TN = 1408	1408

TP, true positive; FP, false positive; TN, true negative; FN, false negative; CRC, colorectal cancer.

**Table 3:** Comparison of screening performance between two quantitative FOB testing methods.

Metric	Centralized Testing Group (OC-Sensor) (95% CI)	POCT-FIT Group (Near-IR) (95% CI)	*p*-Value
Sensitivity	98.9% (95.8–99.9%)	100% (93.2–100%)	–
Specificity	29.2% (27.6–30.8%)	27.3% (26.1–28.5%)	0.001
LR+	1.39 (1.32–1.47)	1.38 (1.31–1.45)	
LR−	0.04 (0.01–0.27)	0 (non-estimable) †	
Youden Index	0.281	0.273	
PPV	3.84%	1.37%	

† LR− is theoretically 0 when sensitivity is 100%; confidence interval is non-estimable. LR+ = sensitivity/(1 − specificity); LR− = (1 − sensitivity)/specificity. The 95% CIs for likelihood ratios were calculated using the delta method with log transformation. *p*-values for sensitivity and specificity were from chi-square tests.

## Data Availability

The data presented in this study are available on request from the corresponding author. The data are not publicly available due to privacy restrictions.

## Abbreviations

CI	Confidence interval
CRC	Colorectal cancer
FIT	Fecal immunochemical testing
FOB	Fecal occult blood
FOBT	Fecal occult blood testing
LR+	Positive likelihood ratio
LR−	Negative likelihood ratio
POCT	Point-of-care testing
POCT-FIT	Point-of-care fecal immunochemical testing
PPV	Positive predictive value
ROC	Receiver operating characteristic
